# Supplementary material for: Genome-Wide Association Analysis for Tuber Dry Matter and Oxidative Browning in Water Yam (Dioscorea alata L.)
Source: Plants (Basel). 2020 Jul 31;9(8):969. doi: 10.3390/plants9080969 (PMC7464735; doi:10.3390/plants9080969)
Supplement: Supplementary file 1 [file plants-09-00969-s001.pdf]

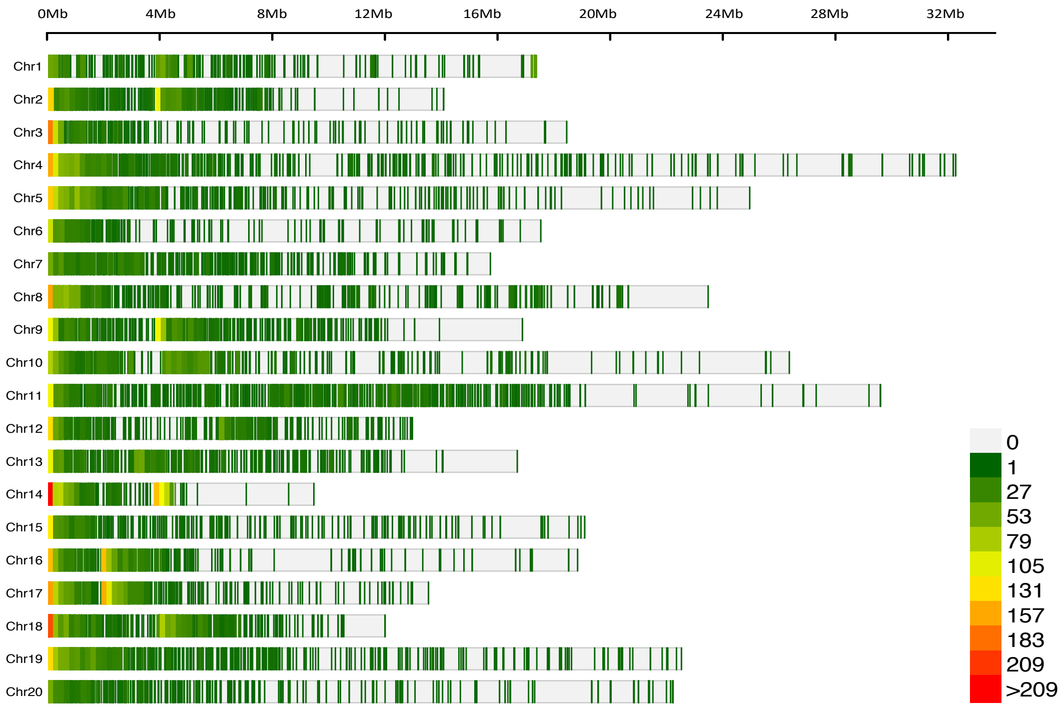


Figure S1: Single nucleotide polymorphism (SNP) distribution and density plot across the *D.alata* reference genome before filtering.

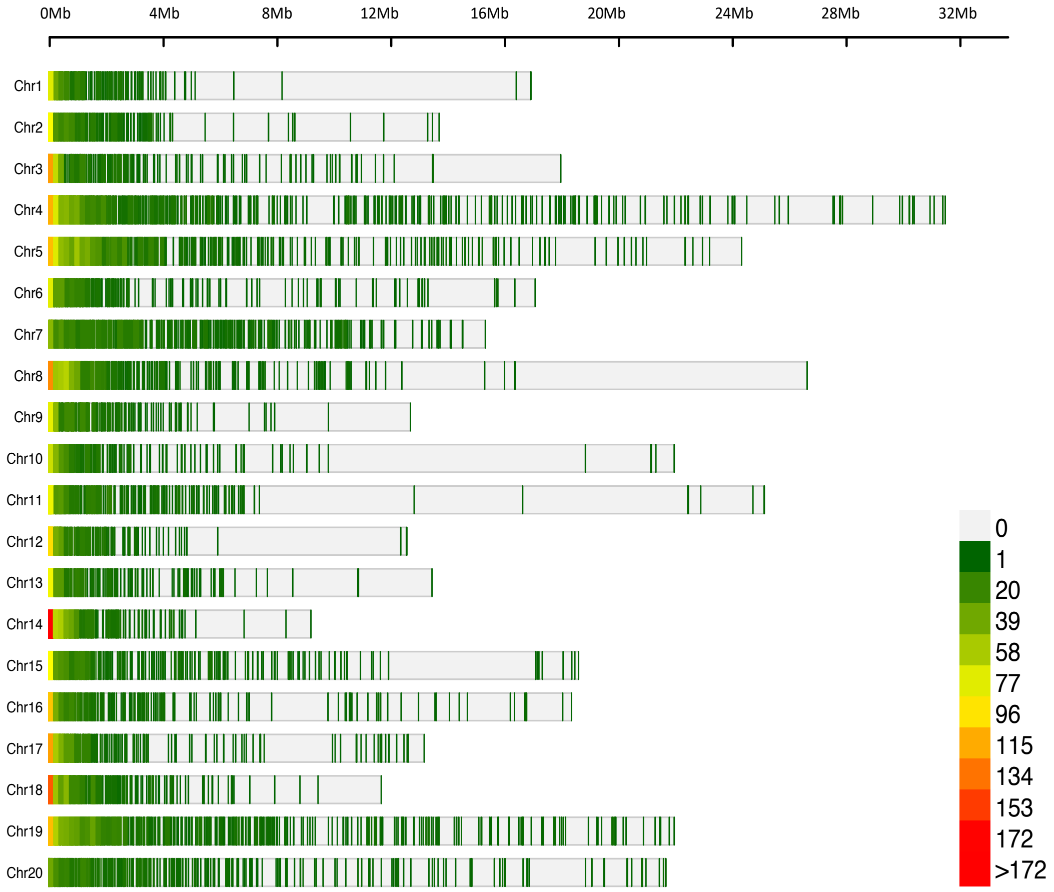


Figure S2: Single nucleotide polymorphism (SNP) distribution and density plot across the *D.alata* reference genome after filtering.
